# Supplementary material for: A case-control neuroimaging investigation of chronic Zika virus-infected adults
Source: bioRxiv. 2025 May 7:2025.03.05.641727. Preprint. [Version 2] doi: 10.1101/2025.03.05.641727 (PMC12247922; doi:10.1101/2025.03.05.641727)
Supplement: 1 [file NIHPP2025.03.05.641727V2-supplement-1.pdf]

bioRxiv preprint doi: https://doi.org/10.1101/2025.03.05.641727; this version posted May 7, 2025. The copyright holder for this preprint (which was not certified by peer review) is the author/funder, who has granted bioRxiv a license to display the preprint in perpetuity. It is made available under aCC-BY 4.0 International license.

Supplementary Materials

Table S1. White matter lesions identified with LPA-LST

ZIKV-CNS-GBS			nonZIKV-GBS			healthy controls		
Cases	TLV (ml)	TNL	Cases	TLV (ml)	TNL	Cases	TLV (ml)	TNL
# 1	0.659	12	# 1	0.835	16	# 1	0.478	7
# 2	1.012	15	# 2	0.773	19	# 2	0.599	13
# 3	0.919	15	# 3	0.769	10	# 3	0.036	2
# 4	0.268	9	# 4	1.628	20	# 4	5.385	42
# 5	0.643	13	# 5	0.786	12	# 5	0.235	5
# 6	0.879	17	# 6	1.012	22	# 6	0.763	9
# 7	0.466	7	# 7	0.567	10	# 7	1.011	9
# 8	0.621	11	# 8	1.582	12	# 8	0.649	11
# 9	0.661	8	# 9	0.911	16	# 9	2.717	16
# 10	0.211	9	# 10	9.307	48	# 10	0.821	14
# 11	0.088	3	# 11	0.573	9	# 11	0.266	2
# 12	1.122	12	# 12	1.029	16	# 12	0.407	13
# 13	1.061	14	# 13	0.126	5	# 13	0.183	6
			# 14	0.831	14			
			# 15	1.851	18			
N cases	Mean (± SD)	Mean (± SD)	N cases	Mean (± SD)	Mean (± SD)	N cases	Mean (± SD)	Mean (± SD)
N = 13	.66 (± .33)	11.15 (± 3.87)	N = 15	1.55 (± 2.28)	16.50 (± 10.24)	N = 13	1.04 (± 1.47)	11.46 (± 10.20)

Note: LPA-LST, Lesion Prediction Algorithm in Lesion Segmentation Toolbox; N cases, Number of cases with FLAIR imaging data; SD, standard deviation; TLV, total white matter lesion volume; TNL, total number of white matter lesions; m.d.; missing data.
